# High-Performance Ethylene Glycol Room-Temperature Gas Sensor Based on Biomass-Derived Na-Doped Porous Carbon Microtubules

**DOI:** 10.3390/nano15221686

**Published:** 2025-11-07

**Authors:** Yan Xu, Qihua Sun, Jialin Li, Zhaofeng Wu, Haiming Duan

**Affiliations:** 1 Xinjiang Key Laboratory of Solid State Physics and Devices, Urumqi 830046, China; 2 School of Physics Science and Technology, Xinjiang University, Urumqi 830046, China; 3 School of Materials Science and Engineering, Xinjiang University, Urumqi 830046, China

**Keywords:** ethylene glycol, gas sensor, Na-doped, biomass, porous carbon microtubules

## Abstract

Ethylene glycol (EG) is a vital industrial raw material. However, it has the potential to be hazardous to the environment and human health. High operating temperatures and long response/recovery times limit the wide application of EG sensors. Thus, we need to develop high-performance room-temperature EG-sensing materials. This paper proposes the direct hydrothermal carbonization of magnolia hair to prepare porous microtubular carbon (CMH) for room-temperature EG sensing. SEM, TEM, and XPS characterization showed that the CMH exhibited a porous microtubular structure and contained Na, which enhanced the adsorption capacity of the CMH for ethylene glycol gas. The CMH sensor exhibits a high response (156.4) to 500 ppm ethylene glycol gas at room temperature with moderate response/recovery time (14.2/37.3 s). It exhibits good linearity in measuring EG gases in the 10–100 ppm range, with a 0.292 ppm theoretical detection limit. Additionally, CMH sensors provide excellent repeatability and long-term stability. The synergistic effect of microtubule porous structure and Na doping is the main reason for enhancing the response of the sensor to EG gas. On this basis, the gas-sensitive enhancement mechanism of CMH was analyzed. The results show that biomass carbon materials provide a new method to prepare high-performance EG gas sensors.

## 1. Introduction

Ethylene glycol (EG) is an industrially important solvent used as an antifreeze agent, coolant, desiccant, and building block in polymers [1]. As a constituent of a wide variety of household products, EG is particularly prevalent in the fields of energy, chemicals, automotive, and textiles [2]. Nevertheless, ethylene glycol is somewhat toxic and when absorbed by the body, it is broken down in the liver into four cytotoxins—glycolaldehyde, glycolic acid, glyoxylic acid, and oxalic acid—which will also have an adverse effect on the central nervous system, heart, lungs, and kidneys [3,4]. Moreover, the volatilization of ethylene glycol gas into the atmosphere can cause air, soil, and water pollution [5]. Therefore, the development of selective, sensitive, cost-effective, and stable ethylene glycol gas sensors is of great importance for human health and the atmospheric environment.

Many scholars have made significant progress in metal oxide semiconductor (MOS)-based ethylene glycol gas sensors. However, MOS sensors also suffer from insufficient stability, poor selectivity, and high operating temperatures, which have seriously hampered their development in various fields. For example, the ternary Au/ZIS/WO_3_ heterostructures synthesized by Li et al. showed an excellent response to 50 ppm ethylene glycol at 200 °C (98.92) [6]. Lilac-like multiple self-supporting WO_3_ nanoneedles developed by Qu et al. exhibited the highest sensitivity (2305) to 100 ppm ethylene glycol at 160 °C [7]. AgCrO_2_ nanoparticles incorporated with magnesium synthesized by Zhu et al. exhibited a high response (16.2–10 ppm ethylene glycol) at an operating temperature of 120 °C [8]. The ErFeO_3_ nanofibers prepared by Wei et al. showed the greatest response (15.8) to 100 ppm ethylene glycol, with an optimum temperature of 230 °C [9]. The BMO/In_2_O_3_ composite synthesized by Zhang et al. produced a response of 38 to 100 ppm ethylene glycol at an operating temperature of 220 °C [10]. In the work by Su et al., a gas sensor based on a hierarchical CuO/Co_3_O_4_ heterostructure showed a high response (6.3) to 100 ppm ethylene glycol at 130 °C [11]. Notwithstanding the advantages of MOS-based EG gas sensors, the high operating temperatures not only cause more energy consumption but also reduce the sensitivity of the sensor and shorten its lifetime. Moreover, EG is highly flammable and poses a risk of gas ignition or explosion [11]. Consequently, there is a significant need to develop new sensor materials capable of detecting ethylene glycol gas at room temperature.

In 2000, a carbon nanotube (CNT)-based gas sensor prepared by Kong et al. achieved detection of the target gas at room temperature, laying the foundation for the application of carbon nanotubes in gas sensors [12]. Subsequently, CNTs-based novel gas sensors have undergone rapid development [13]. However, the preparation of CNTs necessitates harsh conditions and costly equipment, with the materials required for their synthesis comprising non-renewable fossil fuels. This is problematic from both environmental and energy conservation perspectives [14,15]. Therefore, it is important to develop sustainable new carbon-based gas-sensitive materials. In recent years, there has been extensive research and development of biomass due to its advantages of being resourceful, sustainable, environmentally friendly, and inexpensive [16]. Notably, the cylindrical structure of microtubule bundles in plants is perfectly preserved after carbonization, making it an ideal raw material for the preparation of carbon micrometer tubes. On the one hand, the hollow tubular carbon material has a rich porous structure and a larger specific surface area, which provides more active sites for gas adsorption. On the other hand, the hollow tubular structure can provide an effective channel for the diffusion of the target gas [17,18]. Therefore, combining the above advantages, biomass charcoal micrometer tubes are expected to show high performance in ethylene glycol gas detection.

Magnolia (Chinese medicinal name: Xinyi) is a commonly used traditional Chinese medicinal material. Owing to its abundant resources, easy accessibility, low cost, and high cellulose content, it serves as an excellent raw material for preparing carbon materials. Carbonizing magnolia hairs at 300 °C for one hour preserves their original microstructure, yielding a hollow tubular biomass-derived carbon material with abundant pores. This structure provides ample pathways for the adsorption and diffusion of target gases, thereby enhancing the material’s gas-sensing performance. The magnolia hairs contain trace amounts of sodium that can undergo self-doping after carbonization, thereby enhancing the adsorption of target gases by carbon-based materials. Additionally, magnolia hairs are a renewable and naturally biodegradable biomass material. These properties align with current research trends toward sustainable development in the materials field. Its comprehensive advantages in microstructure, composition, resource economy, and environmental friendliness make it an ideal precursor for carbon-based gas-sensitive materials in this study, providing effective support for the preparation of economically viable, environmentally friendly, gas-sensitive materials capable of detecting target gases at room temperature.

In this study, original magnolia hairs (OMH) were prepared as a biomass carbon material with a porous microtubular structure by a simple hydrothermal carbonization method. For the first time, they were used to detect ethylene glycol gas at room temperature. The carbonized magnolia hairs (CMH) retained the original tubular structure and exhibited high sensitivity and selectivity, as well as good long-term stability and repeatability for glycol gas (500 ppm) at room temperature. It is noteworthy that this work presents, for the first time, the application of used Magnolia herb as a biomimetic material for gas sensors. This study provides new tactics for the preparation of gas-sensitive sensing materials by directly using the natural structure of biomass.

## 2. Materials and Methods

### 2.1. Materials and Reagents

NH_3_ (25 wt%), aniline (C_6_H_7_N (99.9 wt%)), acetone (C_3_H_6_O (99.5 wt%)), formaldehyde (CH_2_O (37 wt%)), trimethylamine (C_3_H_9_N (33 wt%)), toluene (C_7_H_8_ (99.5 wt%)), methanol (CH_3_OH (99.9 wt%)) and ethylene glycol ((CH_2_OH)_2_ (99 wt%)) were purchased from Sinopharm Chemical Reagent Co., Ltd., Beijing, China. Hydrogen peroxide (H_2_O_2_ (30 wt%)) was purchased from Aladdin Reagent Co., Ltd., Shanghai, China. Xinyi Flower Herbs from Pharmacy (Urumqi, China).

### 2.2. Preparation of CMH Sample

The hairs on the outer surface of the magnolia bracts were meticulously removed and rinsed with deionized water. The clipped hairs were then placed into the PTFE liner; deionized water was added and stirred. After stirring well, the collected product was held in a hydrothermal autoclave reactor at 180 °C for 12 h. When the reaction was completed, the product was collected and rinsed twice with deionized water. Subsequently, it was put into a drying oven for the drying process. Finally, the dried samples were placed in a chemical meteorological deposition furnace (heating rate of 5 °C/min) and carbonized at 300 °C for 1 h. At the end of the reaction, after cooling to room temperature, the samples were removed and named CMH.

### 2.3. Materials Characterization and Tests

The morphology and microstructure were characterized by SEM ((FE-SEM, Zeiss, Sigma300, Oberkochen, Germany) and TEM (TEM, JEOL, JEM 2100 F, Tokyo, Japan), respectively. Energy-dispersive X-ray spectroscopy (EDS, S-4800, Hitachi, Japan) was utilized to investigate the chemical element composition, and the surface species and chemical states were analyzed by XPS (XPS, ESCALAB 250Xi, Waltham, MA, USA). The infrared spectrum was characterized using FTIR (FT-IR, Bruker, VERTEX 70 RAMI, Ettlingen, Germany). The structure and composition of the samples were analyzed via X-ray diffraction (XRD, Rigaku, D8 Advance, Tokyo, Japan, with Cu-Kα radiation). The absorbance (200–800 nm) of the samples was tested using UV-Vis spectroscopy (UV-Vis, Lambda 650, Waltham, MA, USA). The reaction products were measured using in situ infrared spectroscopy instrumentation (INVENIO, Bruker, Ettlingen, Germany). The gas-sensing test was conducted using a CGS-MT gas-sensing measurement system (Beijing Sinoagg Co., Ltd., Beijing, China).

### 2.4. Preparation and Sensing Test of CMH Sensors

The carbonized hairs of magnolia (CMH) were placed in agate mortar with a few drops of deionized water and were lightly ground. The abrasive solution was then spread evenly (approximately 200 μm) onto the electrode pads using a brush and placed in a sample box to dry naturally at room temperature (25 °C) for 24 h. Following the conclusion of the drying process, the gas-sensing performance was subjected to testing at ambient temperature. This testing was conducted utilizing the multifunctional probe station (CGS-MT).

The CGS-MT gas measurement system contains a static gas distribution chamber with a capacity of 1 L. The chamber contains an evaporation dish for the injection of the target gas. It contains two fans on either side to quickly generate the target gas in the chamber. The test platform within the chamber is equipped with four probes. During the test, the electrode pads coated with sensing material were placed on the test bench, and two probes of the same test channel were connected to the electrodes at both ends of the electrode pads. The test voltage was set to 4 V and the current of the gas sensor was recorded using the I-t measurement mode. The temperature of the evaporating dish was set to 200 °C (the boiling point of ethylene glycol is 197.3 °C), and a corresponding volume of 99 wt% ethylene glycol liquid was drawn with a microsampler and injected onto the evaporating dish. The change in the current signal of the gas sensor to the target gas was then recorded after the liquid evaporated. The gas distribution equation for the target gas is shown in (1):

(1) $$V_{solution}=\frac{V_{chamber}\cdot M_{solute}\cdot C\cdot P}{\varphi_{solution}\cdot \rho_{solution}\cdot R\cdot T}$$

where *V_solution_* denotes the volume of the liquid to be injected; *V_chamber_* is the volume of the chamber (1 L); *M_solute_* is the molecular weight of the solute (ethylene glycol: 0.06207 kg/mol; trimethylamine: 0.05911 kg/mol; hydrogen peroxide: 0.03401 kg/mol; ammonia: 0.01703 kg/mol; formaldehyde: 0.03003 kg/mol; aniline: 0.09313 kg/mol; acetone: 0.05808 kg/mol; toluene: 0.09214 kg/mol; methanol: 0.03204 kg/mol); C is the concentration of the target gas (ppm); P is the pressure in the test chamber (1.013 × 10^5^ Pa); *φ_solution_* is the purity of the solute (refer to Section 2.1); *ρ_solution_* is the density of the solution (ethylene glycol: 1110 kg/m^3^; trimethylamine: 880 kg/m^3^; hydrogen peroxide: 1130 kg/m^3^; ammonia: 910 kg/m^3^; formaldehyde: 820 kg/m^3^; aniline: 1020 kg/m^3^; acetone: 790 kg/m^3^; toluene: 870 kg/m^3^; methanol: 790 kg/m^3^); R is the molar gas constant (8.31 J/mol∙K); T is the ambient temperature of the test chamber (298.15 K).

The response value is defined as $Response=I_{g}/I_{a}$ ($I_{g}$ and $I_{a}$ are the currents of the sensor in the target gas and air, respectively). The response time is defined as the time required to reach 90% of the saturated response value, and the recovery time is defined as the time required to return to 10% of the initial response value.

## 3. Results and Discussion

### 3.1. Characterization Results

**Figure 1:**
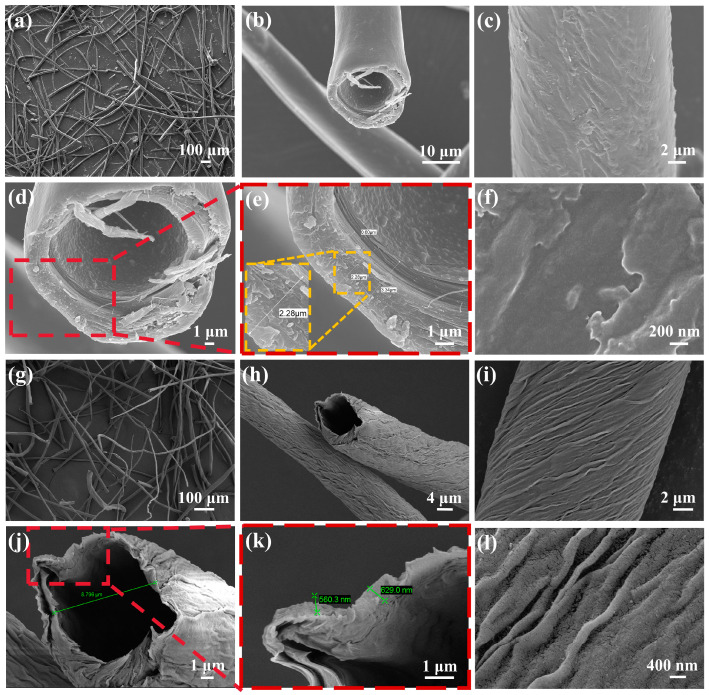
SEM images of OMH (**a**–**f**) and CMH (**g**–**l**).

The OMH and CMH samples were characterized by SEM. As illustrated in Figure 1a–f, OMH displayed a hollow structure in the cross sections. The mean diameter of magnolia hairs was approximately 20 μm and the thickness of the tube wall was approximately 2–3 μm. As shown in Figure 1g–l, after carbonization at 300 °C, CMH retains a hollow tubular structure, the average diameter becomes about 9 μm, and the tube wall shrinks to about 0.6 μm. Their surface exhibited a spiral configuration. The hollow structure enhanced the specific surface area of the CMH, facilitating the creation of more channels and reaction sites for the target gas, which was beneficial to improve the sensitivity and the sensing speed [15].

**Figure 2:**
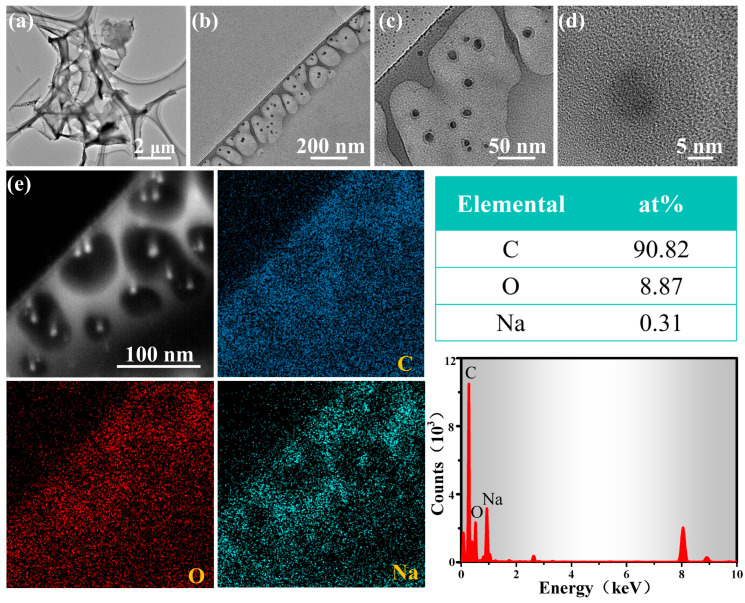
(**a**–**d**) TEM image of CMH; (**e**) EDS elemental mapping of CMH and C, O, and Na contents.

To gain a deeper understanding of the structure as well as the elemental composition of CMH, further characterization was conducted using TEM. The higher magnification images of the CMH (Figure 2b,c) reveal the presence of numerous pores, with an average diameter of approximately 10 nm (Figure 2d). These pores increase the reaction area of the material, providing more active me i points for the gas while also promoting the diffusion of the gas through the sensing material, which helps in the adsorption and desorption of the gas molecules [9]. We chose one of the CMH microtubules to characterize its elemental distribution. From Figure 2e, the elements C, O, and Na are uniformly dispersed on the CMH microtubule. The average contents of C, O, and Na were determined to be 90.82%, 8.87% and 0.31%, respectively.

**Figure 3:**
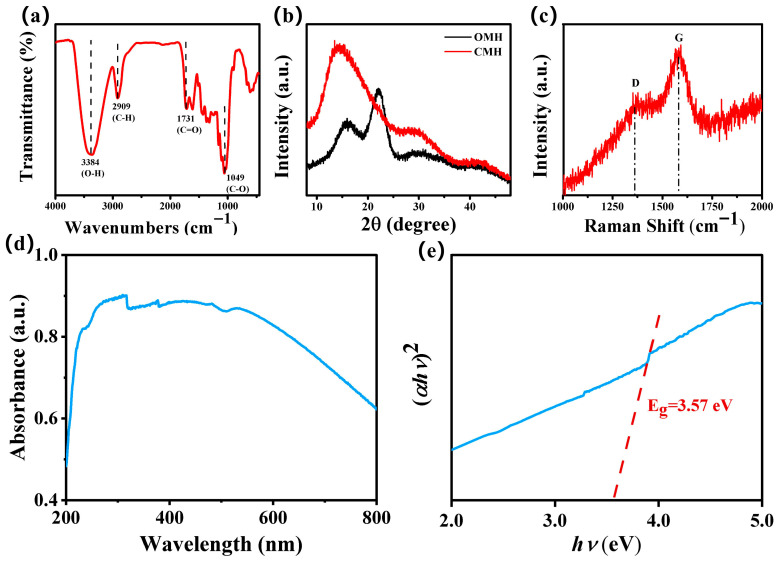
(**a**) FTIR spectra; (**b**) XRD spectrum; (**c**) Raman spectra; (**d**) UV-Vis spectra; and (**e**) Tauc plots of CMH.

The functional groups of CMH were analyzed using FTIR spectroscopy (Figure 3a). The peak at 3384 cm^−1^ is associated with the stretching and bending vibrations of the O-H group [19,20]. The CMH peaks near 2909 cm^−1^ and 1731 cm^−1^ are attributed to C-H and C=O groups, respectively [21]. The peak near 1058 cm^−1^ is attributed to the vibration of the C-O group [22]. The structural properties of OMH and CMH were characterized using XRD and the results are shown in Figure 3b. The characteristic peaks of CMH at 16.1° and 22.2° correspond to the (101) and (002) crystal planes of cellulose, respectively [23]. After carbonization, the characteristic diffraction peak around 14.2° for CMH corresponds to the characteristic peak of carbon materials [24]. The Raman spectra showed two peaks at 1361 cm^−1^ and 1582 cm^−1^, corresponding to the D-band (sp3-type disordered carbon) and G-band (sp2-type ordered graphitic carbon) of carbon, respectively (Figure 3c) [25,26]. The UV spectra of the sample were measured using a UV-Vis spectrophotometer (Figure 3d). The processing of data through the relationship between the optical bandgap and the absorption coefficient yields Figure 3e. The results show that the bandgap of CMH was 3.57 eV, which proves the semiconductor property of CMH.

**Figure 4:**
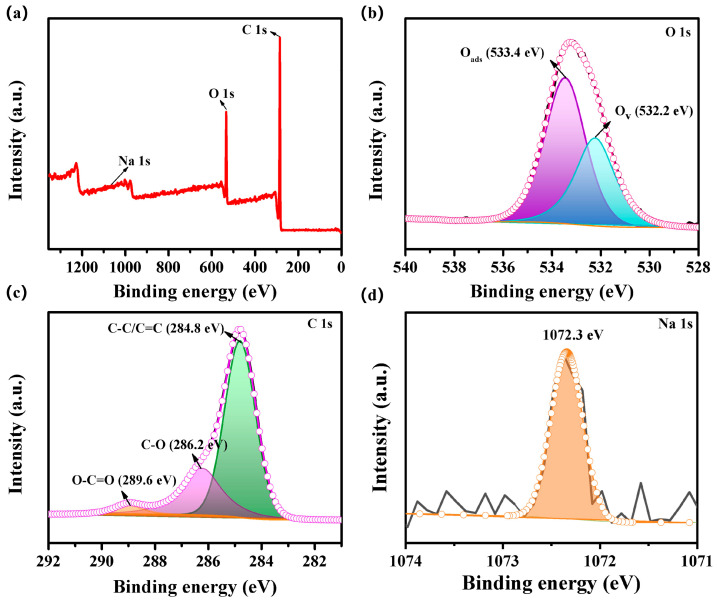
(**a**) XPS survey spectra; (**b**) O 1s spectra; (**c**) C 1s spectra; and (**d**) Na 1s spectra.

Figure 4a shows the survey spectra of the CMH sample, which demonstrates that the elements (C, O, Na) in the samples are consistent with those in the EDS analysis. The characteristic peaks at 285.1 eV and 532.9 eV correspond to C 1s and O 1s, respectively, and a peak at 1072.3 eV appears as Na 1s. Figure 3b shows the high-resolution spectra of the CMH material O 1s. The two characteristic peaks of CMH at 532.2 eV and 533.4 eV correspond to oxygen vacancy (O_v_) and adsorbed oxygen (O_ads_), respectively [6,7]. In general, oxygen vacancies and absorbed oxygen have a major impact on the gas-sensitive performance [27,28]. Adsorbed oxygen has been demonstrated to react directly with the target gas. The presence of oxygen vacancies within the sensing materials can provide additional active sites, thereby facilitating the adsorption of oxygen clusters onto the material’s surface and enhancing the sensor’s response to ethylene glycol gas. Consequently, the presence of these two oxygen species exerts an influence on the gas sensitivity. The peaks at 284.8 eV, 286.2 eV, and 289.6 eV in Figure 3c represent C-C/C=C, C-O, and O-C=O functional groups, respectively [25,29]. The high-resolution spectrum of Na elements for the CMH sample is shown in Figure 4d, with the XPS peak at 1072.3 eV [30].

### 3.2. Gas-Sensing Properties

**Figure 5:**
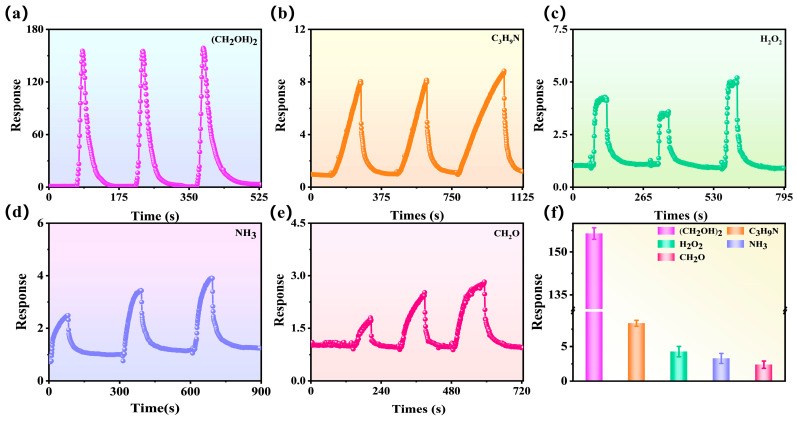
(**a**–**e**) Sensing curves of the CMH towards 500 ppm (CH_2_OH)_2,_ C_3_H_9_N, H_2_O_2_, NH_3,_ and CH_2_O at room temperature (25 °C). (**f**) Average response.

To further investigate the gas-sensitive performance of the CMH sensor, the responses of 500 ppm NH_3_, C_6_H_7_N, C_3_H_6_O, CH_2_O, C_3_H_9_N, C_7_H_8_, CH_3_OH, (CH_2_OH)_2_, and H_2_O_2_ were tested at room temperature (25 °C), and the results are shown in Figure 5. As demonstrated by the response curve, the response of CMH to the reducing gas is upward, thus indicating the inductive properties of n-type semiconductors. Figure 5a demonstrates the ultra-high sensitivity of the CMH sensor to 500 ppm (CH_2_OH)_2_, with three stable cycles exhibiting good repeatability. The detection of several common interfering gases (C_3_H_9_N, H_2_O_2_, NH_3,_ CH_2_O) (Figure 5b,c) shows that the CMH sensor response to them is much smaller than to (CH_2_OH)_2_, which indicates that the CMH has good immunity to interference. The CMH sensor was almost unresponsive to other gases (C_6_H_7_N, C_3_H_6_O, C_7_H_8_, CH_3_OH) (Figure S1 in the Supplementary Materials). As demonstrated in Figure 5f, the statistical CMH sensor response to (CH_2_OH)_2_ has a mean value of 156.4 (with a standard deviation of 2.02), the response to C_3_H_9_N has a mean value of 8.3 (with a standard deviation of 0.74), the response to H_2_O_2_ has a mean value of 4.3 (with a standard deviation of 0.74), the response to NH_3_ has a mean value of 3.3 (with a standard deviation of 0.73), the response to CH_2_O has a mean value of 2.4 (with a standard deviation of 0.53), and the response to NH_3_ has a mean value of 2.3 (with a standard deviation of 0.73). The mean value of CH_2_O is 2.4 (standard deviation 0.53). In summary, the sensor response to (CH_2_OH)_2_ was 19–65 times higher than that of other gases. This is a more intuitive demonstration of the good selectivity of CMH sensors.

**Figure 6:**
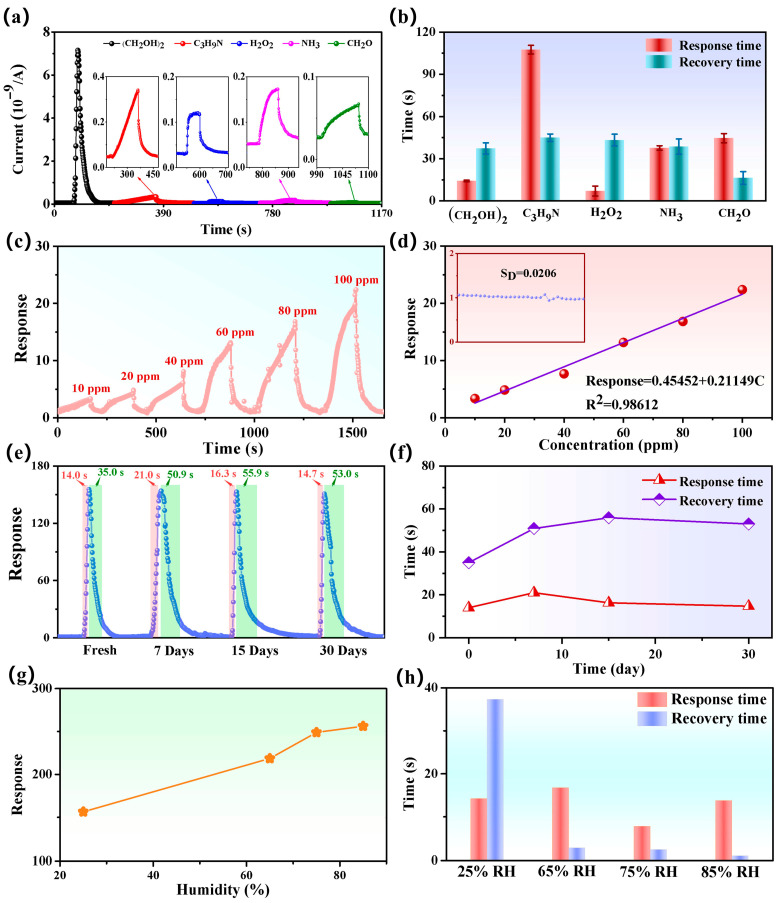
(**a**) Sensor current response in different gases (500 ppm (CH_2_OH)_2_, C_3_H_9_N, H_2_O_2_, NH_3_, CH_2_O); (**b**) Response time and recovery time statistics chart; (**c**) the response curves of CMH to different concentrations (10–100 ppm) of (CH_2_OH)_2_ at room temperature (25 °C); (**d**) linear fitting relationship between response and concentration; (**e**) long-term stability response curve and response/recovery time of CMH to 500 ppm (CH_2_OH)_2_; (**f**) long-term stability line chart under different humidity conditions; (**g**) the CMH sensor response values for 500 ppm ethylene glycol gas; (**h**) response (recovery) times.

Figure 6a visually shows that the CMH sensor exhibits a substantially higher response value for ethylene glycol gas in comparison to other gases (C_3_H_9_N, H_2_O_2_, NH_3_, CH_2_O), which reflects the excellent selectivity of the sensor for ethylene glycol gas. Figure S2 (in the Supplementary Materials) shows the response and response/recovery time of the CMH sensor for various gases, such as (CH_2_OH)_2_ (13.5 s/33.0 s), C_3_H_9_N (102.8 s/47.5 s), H_2_O_2_ (10.0 s/43.0 s), NH_3_ (36.9 s/39.0 s), and CH_2_O (45.9 s/21.0 s). CMH demonstrated the shortest response time to H_2_O_2_ (10.0 s), the longest response time to C_3_H_9_N (102.8 s), the shortest recovery time to CH_2_O, and the longest recovery time to C_3_H_9_N (47.5 s). Figure 6b shows the statistics of response time and recovery time, and the average response/recovery time of the CMH sensor for each gas is (CH_2_OH)_2_ (14.2 s/37.3 s), C_3_H_9_N (107.6 s/44.9 s), H_2_O_2_ (9.0 s/43.3 s), NH_3_ (37.6 s/38.6 s), and CH_2_O (44.6 s/16.3 s). The response time and recovery time of the sensor for (CH_2_OH)_2_, although not the shortest, can complete a cycle period in 51.5 s on average. Comparing the cycle periods of the other gases—C_3_H_9_N (152.5 s), H_2_O_2_ (52.3 s), NH_3_ (76.2 s), CH_2_O (60.9 s), and (CH_2_OH)_2_—this has the shortest cycle period. This further shows the potential of preparing biomass gas-sensitive materials from magnolia flowers for glycol gas detection.

Ethylene glycol is a toxic gas that is distributed extensively in everyday life, being present in a variety of products, including pharmaceuticals, personal care products, coolants, and detergents. Long-term exposure to high concentrations of ethylene glycol gas can impair liver and kidney function. Short-term human exposure to ethylene glycol gas should not exceed 14.4 ppm [8]. Therefore, further evaluate the limit of detection (*LoD*) of the CMH sensor. The *LoD* of the theoretical calculation is defined as $LoD={3S}_{D}/m$, where m is the slope of the linear part of the calibration curve and $S_{D}$ is the standard deviation of noise in the response curve. To evaluate the *LoD* of the CMH sensor, the response curves of the CMH sensor to different concentrations of ethylene glycol gas were tested. As shown in Figure 6c,d, the response of the CMH sensor to ethylene glycol gas increases gradually with increasing gas concentration (10–100 ppm). From Figure 6d, the response of the CMH sensor shows a good linear relationship with the concentration of ethylene glycol gas. According to the noise data of the CMH sensor in Figure 6d (inset), the values of $S_{D}$ are approximately 0.0206 and m is approximately 0.21149. The theoretically calculated *LoD* is about 0.292 ppm. This indicates that the CMH sensor has high sensitivity to ethylene glycol gas.

Furthermore, the long-term stability of CMH was tested to provide a more comprehensive evaluation of its sensing performance. As demonstrated in Figure 6e, following 7, 15, and 30 days, the response of CMH to 500 ppm (CH_2_OH)_2_ was 153.9, 152.9, and 150.8, respectively, with a decrease of 0.96%, 1.61%, and 2.96% in response. The CMH sensor exhibits excellent long-term stability. In addition, the response/recovery times labeled in Figure 6e are Fresh (14.0 s/35.0 s), 7 days (21.0 s/50.9 s), 15 days (16.3 s/55.9 s), and 30 days (14.7 s/53.0 s). As illustrated in Figure 6f, the response time of the CMH sensor remains relatively stable as the number of days increases. However, a significant increase in recovery time is observed after seven days. The recovery performance of the CMH sensor requires enhancement.

Relative humidity (RH) is an indispensable condition for sensor applications. Thus, the effect of humidity on sensor sensitivity was studied by exposing the sensor to 500 ppm of ethylene glycol at different humidity levels (65% RH, 75% RH, and 85% RH). The corresponding response versus relative humidity is plotted in Figure 6g. Under high-humidity conditions, the response increases as the humidity increases. The reasons for this are that water vapor forms hydroxyl groups (OH^-^) and hydrogen ions (H_3_O^+^) on the surface of the CMH-sensing materials, and H_3_O^+^ can bind to ethylene glycol to form hydrogen bonds, thus enhancing the sensor’s response to ethylene glycol gas [31,32]. In Figure 6h, the response recovery time is significantly shorter at higher humidity levels. This is possibly because ethylene glycol only forms hydrogen bonds with H_3_O^+^ ions, which are not very stable. When there is an excess OH^−^ ions, H_2_O is generated and ethylene glycol is desorbed [33]. These experimental results further validate the stability performance of the CMH gas sensor in room-temperature environments and humidity-enhancement conditions, laying the foundation for the practical application of this sensor in high-humidity scenarios.

Table 1 summarizes the last three years of research on ethylene glycol gas sensors of different materials. The table reveals problems with most of the sensors, such as high optimal operating temperatures and slow adsorption and desorption rates. In comparison, the CMH has a definite advantage in terms of operating temperature, enabling ethylene glycol gas detection at room temperature. In addition, a comparison of key performance metrics reveals that the CMH sensor exhibits a shorter recovery time and a lower *LoD* than other materials. These combined results reaffirm the potential of biomass-derived sensing materials, prepared from OMH, for ethylene glycol gas detection.

### 3.3. Gas-Sensing Mechanism

The primary mechanisms through which semiconductor gas sensors interact with target gases are adsorption and desorption mechanisms. When the sensor is exposed to air, due to the relatively large electronegativity of oxygen, the oxygen ions in the air will be adsorbed on the surface of the sensor, seizing the electrons on the surface of the sensing material and forming $O_{2}^{-}$ ions, as shown in Equations (1) and (2) [37].

(2) $$O_{2}(gas)↔O_{2}(ads)$$

(3) $$O_{2}\left(ads\right)+e^{-}↔o_{2}^{-}(ads)$$

**Figure 7:**
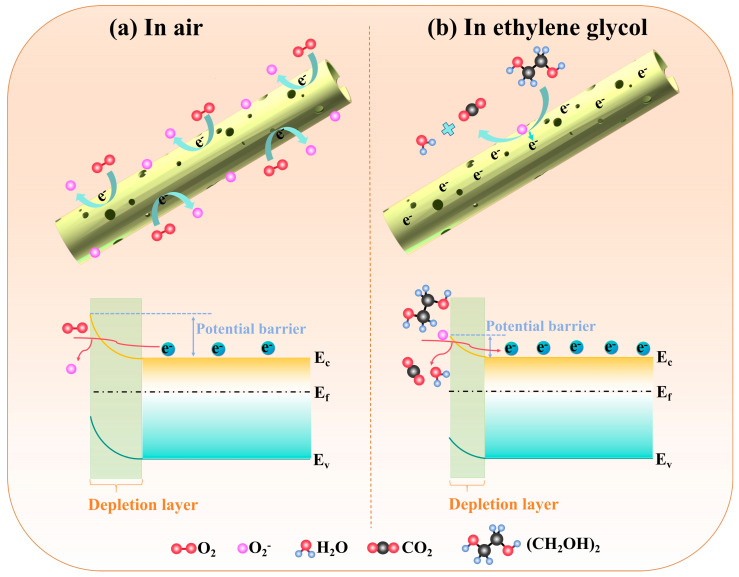
The interaction between (CH_2_OH)_2_ molecule and the CMH, and the detection mechanism of CMH for ethylene glycol gas.

Firstly, from the response curves in Figure 5a–f, the response of the CMH to the reducing gas is upward, demonstrating the inductive properties of n-type semiconductors. Thus, when the CMH sensor is placed in the air (Figure 7a), the oxygen in the air captures electrons from the surface of the CMH and converts them to oxygen-negative ions, and the surface electron concentration of the material decreases, forming an electron depletion layer and increasing the sensor resistance. When the CMH was exposed to ethylene glycol gas (Figure 7b), the reaction between the gas molecules and the surface oxygen-negative ions occurred. The adsorbed oxygen ions released the trapped electrons back into the conduction band of the sensing material, reducing the thickness of the electron-depletion layer and decreasing the material’s resistance. The above reaction is shown in the following equation [5]:

(4) $$2{\left(CH_{2}OH\right)}_{2}+5o_{2}^{-}\left(ads\right)\to 4CO_{2}+6H_{2}O+5e^{-}$$

Secondly, CMH exhibits a nanoscale porous microtubular structure (Figure 2), which provides abundant active sites to improve its sensitivity to ethylene glycol gas. Beyond the material microstructure, the charge depletion layer (L) is another crucial factor affecting the sensing performance of chemoresistive gas sensors [21]. As derived from Equation (5), L is inversely proportional to the carrier concentration (Nd) of the sensing material, meaning that reducing Nd contributes to increasing L and thereby enhancing sensitivity.

(5) $$L\propto \frac{N_{t}}{N_{d}}$$

Characterization results from EDS (Figure 2e) and XPS (Figure 4d) confirm that the CMH sensing material is self-doped with sodium (Na). Typically an n-type doping, Na facilitates electron transfer from sodium to carbon material. These electrons neutralize part of the holes, which reduces the carrier concentration of the carbon material and further enhances the sensor’s sensitivity. Additionally, first-principles calculations suggest that Na doping can enhance the material’s adsorption energy toward target gases, providing an additional boost to sensitivity [38,39]. In summary, the porous microtubular structure and Na doping exert a synergistic effect, which enhances the gas-sensing performance of CMH for ethylene glycol detection. Consequently, the CMH sensor demonstrates superior gas-sensing properties.

**Figure 8:**
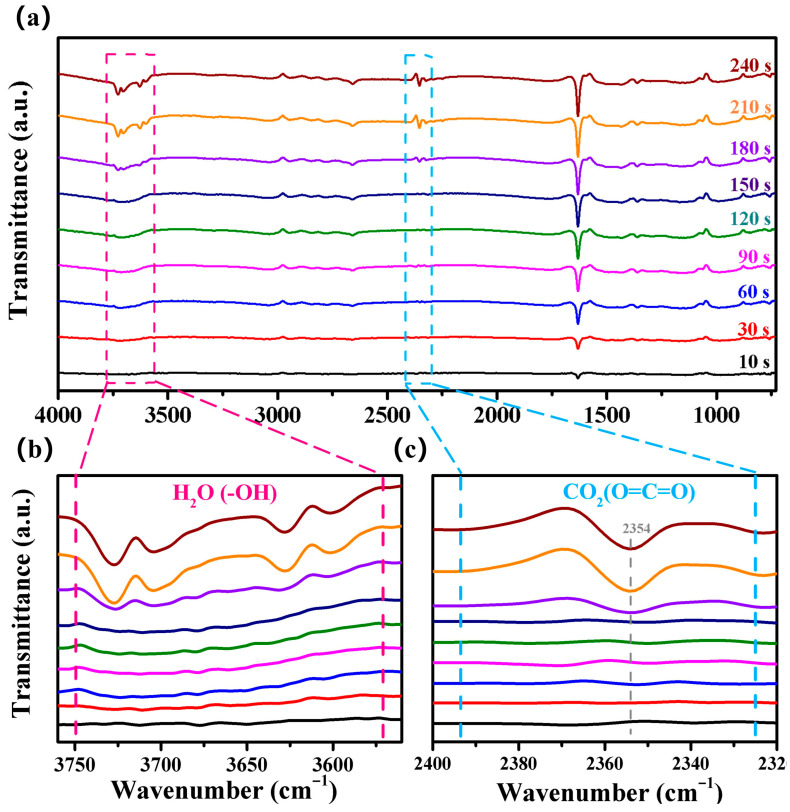
(**a**–**c**) Time-resolved in situ FTIR spectra of the response processes of the CMH sensor exposed to ethylene glycol gas at 25 °C/25% RH.

To conduct a more in-depth investigation into the sensing process of the CMH sensor, an in situ Fourier transform infrared (FTIR) spectroscopy of CMH exposed to ethylene glycol gas was performed. In Figure 8a, the curves illustrate the in situ FTIR spectrum of the CMH upon exposure to varying times. in Figure 8b. The signal peaks at 3570–3750 cm^−1^ are attributed to the free -OH stretching in H_2_O [40,41]. The dual peak centered around 2354 cm^−1^ in Figure 8c is related to O=C=O asymmetric stretching in CO_2_ [42,43,44]. Additionally, the intensities of these peaks gradually increase with prolonged exposure to ethylene glycol gas. The increasing signals of -OH and O=C=O further provide evidence that ethylene glycol decomposes into H_2_O and CO_2_ (the ethylene glycol reaction products are consistent with Equation (4)).

## 4. Conclusions

In this work, porous CMH with microtubular structures was successfully prepared using a simple and environmentally friendly method of carbonization. The gas-sensitive properties of CMH sensors were tested in detail at room temperature. The results show that the CMH sensor response higher to ethylene glycol gas at 500 ppm is 19–65 times higher than its response to other interfering gases, with a response/recovery time of 14.2 s/37.3 s. Self-doping of Na was realized by using the Na element contained in Magnolia, which improves the adsorption capacity of the CMH to ethylene glycol gas. The ethylene glycol gas sensor exhibits good selectivity, repeatability, and long-term stability. It is important to note that the detection of ethylene glycol gas is possible at room temperature. By analyzing the sensing mechanism of CMH, it was found that the combination of a microtubular structure, a porous structure, and Na doping played a synergistic sensitizing role in ethylene glycol gas detection. This work provides a new idea through a simple, cost-effective, and green approach to preparing biomass-sensing materials.

## Figures and Tables

**Table 1:** Comparison of ethylene glycol gas sensors of various materials reported in the recent literature.

Sensing Materials	Temp. (°C)	Con. (ppm)	Res. (R_a_/R_g_)	*T*_res_/*T*_rec_ (s)	*LoD* (ppm)	Ref.
ZnO/rGO	220	100	277	38/26	1 ^a^	[33]
ErFeO_3_	230	100	15.8	61/39	0.035 ^b^	[9]
BMO/In_2_O_3_	220	100	38	26/129	1 ^a^	[10]
SmFeO_3_	180	100	117	80/95	5 ^a^	[34]
In_2_O_3_@ZnO	200	200	200.12	53/50	1 ^a^	[5]
AgCrO_2_	120	10	16.2	32/130	1 ^a^	[8]
ZnO/ZnCo_2_O_4_	160	100	15.63	190/45	1.59 ^b^	[35]
WO_3_	160	100	2305	107/215	1 ^a^	[7]
Au/ZnIn_2_S_4_/WO_3_	220	50	98.92	57/136	0.006 ^b^	[6]
La-doped ZnSnO_3_	140	100	1488.79	—	0.2 ^a^	[28]
Sm^3+^ doped Bi_2_MoO_6_	240	100	60.1	20/78	5 ^a^	[36]
CuO/Co_3_O_4_	130	100	6.3	33/54	5 ^a^	[11]
Ag decorated CuGaO_2_	165	10	3.46	15/30	0.067 ^b^	[3]
CMH	25	100	22.4 ^c^	135.6/56.9	0.292 ^b^	This work
		500	155.4 ^c^	14.7/37.3		

^a^. Experimental; ^b^. theoretical; ^c^. I_a_/I_g._

## Data Availability

The data presented in this study are available from the corresponding authors upon reasonable request.

## Supplementary Materials

The following supporting information can be downloaded at: https://www.mdpi.com/article/10.3390/nano15221686/s1, Figure S1: Sensing curves of the CMH towards 500 ppm (a) C_6_H_7_N, (b) C_3_H_6_O, (c) C_7_H_8_, and (d) CH_3_OH at room temperature.; Figure S2: Response/recovery curves in 500 ppm (CH_2_OH)_2_, C_3_H_9_N, H_2_O_2_, NH_3_, and CH_2_O; Figure S3: Response at different relative humidity environments to 500 ppm of ethylene glycol.
