# Supplementary material for: High-Performance Ethylene Glycol Room-Temperature Gas Sensor Based on Biomass-Derived Na-Doped Porous Carbon Microtubules
Source: Nanomaterials (Basel). 2025 Nov 7;15(22):1686. doi: 10.3390/nano15221686 (PMC12655541; doi:10.3390/nano15221686)
Supplement: Supplementary file 1 [file nanomaterials-15-01686-s001.zip › nanomaterials-3951286-supplementary.pdf]

Supplementary Material

High-performance ethylene glycol room temperature gas sensor based on biomass-derived Na-doped porous carbon microtubules

Yan Xu[1,2], Qihua Sun[1,2*], Jialin Li[1,2], Zhaofeng Wu[1,3*], Haiming Duan[1,2*]

[1] *Xinjiang Key Laboratory of Solid State Physics and Devices, Urumqi 830046, China*

[2] *School of Physics Science and Technology, Xinjiang University, Urumqi 830046, China*

[3] *School of Materials Science and Engineering, Xinjiang University, Urumqi 830046, China*

E-mail: sunqh@xju.edu.cn (Q. S.),wuzf@xju.edu.cn (Z. W.), dhm@xju.edu.cn (H. D.)

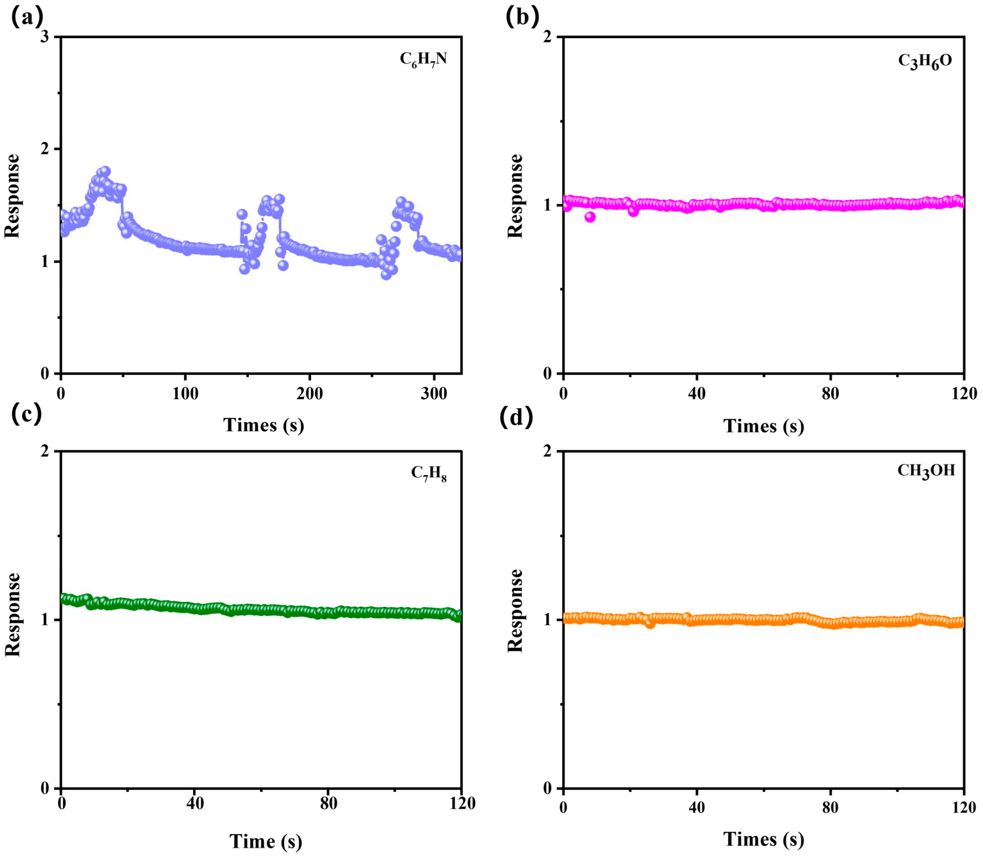

Figure S1 Sensing curves of the CMH towards 500 ppm(a) C_6H_7N, (b) C_3H_6O, (c)C_7H_8, (d) CH_3OH at room temperature.

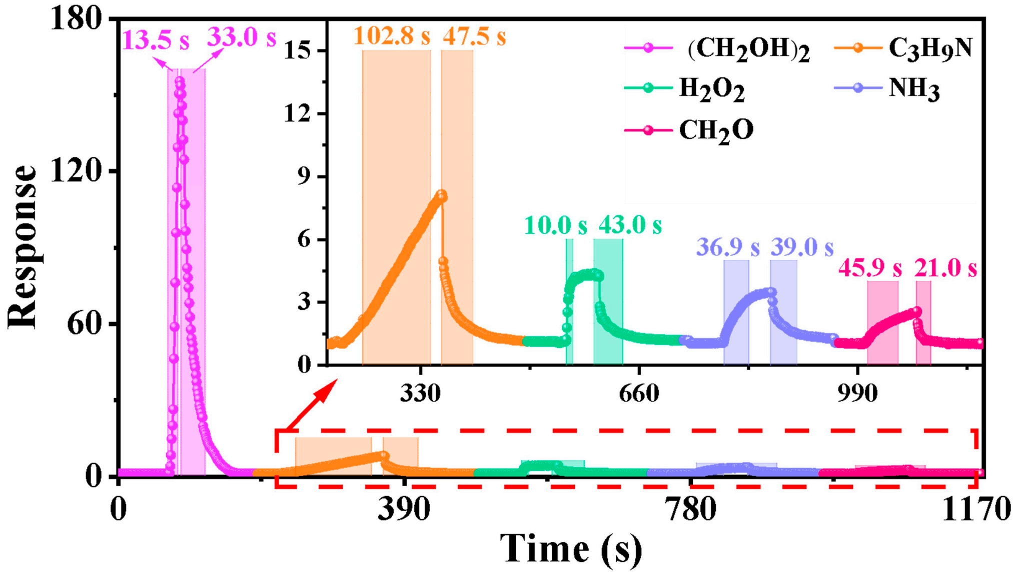

Figure S2 Response/recovery curves in 500 ppm $(CH_2OH)_2$, C_3H_9N, H_2O_2, NH_3, CH_2O.

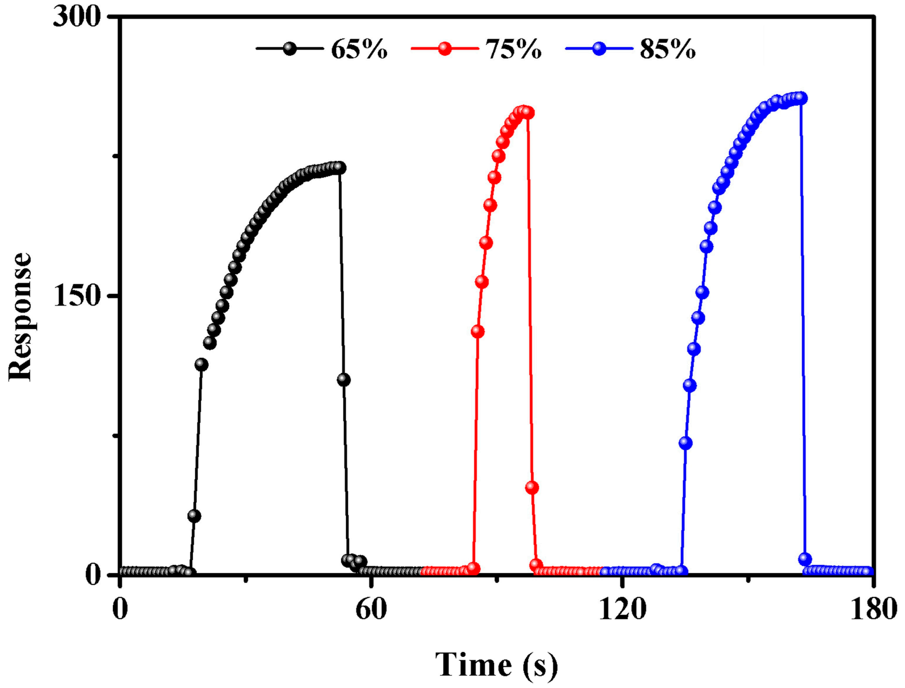

Figure S3 Response at different relative humidity environments to 500 ppm of ethylene glycol.
